# A hepatoid carcinoma of the pancreatic head

**DOI:** 10.1186/s40792-016-0197-7

**Published:** 2016-08-03

**Authors:** D. Stamatova, L. Theilmann, C. Spiegelberg

**Affiliations:** 1 HELIOS Hospital Pforzheim, Kanzlerstrasse 2 – 7, Pforzheim, 75175 Germany; 2 Department of Surgery, HELIOS Hospital Pforzheim, Kanzlerstrasse 2 – 7, Pforzheim, 75175 Germany; 3 Department of Gastroenterology, HELIOS Hospital Pforzheim, Kanzlerstrasse 2 – 7, Pforzheim, 75175 Germany; 4 Institute of Pathology, HELIOS Hospital Pforzheim, Kanzlerstrasse 2 – 7, Pforzheim, 75175 Germany

**Keywords:** Pancreas, Neoplasm, Hepatoid, Retroperitoneal, Rare

## Abstract

Hepatoid carcinoma (HC) is an extremely rare form of neoplasm. Its cellular structure resembles that of a hepatocellular carcinoma (HCC). To date, only 26 cases of hepatoid carcinoma of the pancreas have been reported in the literature. We report the diagnosis of a hepatoid carcinoma of the pancreatic head in a 78-year-old male patient. The tumor was detected incidentally during routine abdominal ultrasound scanning. Laboratory tests did not show any abnormalities except for a monoclonal gammopathy of undetermined significance. After CT, MRI, and laparoscopic biopsy that failed to obtain the diagnosis, the patient underwent a Whipple procedure. The final pathology report described a hepatoid carcinoma of the pancreatic head (pathological T3, N0 (0/10), L0, V0, R0, M0). After the patient recovered, no further therapy was recommended by the tumor board and he was discharged. Regular follow-up was suggested; however, the patient suddenly died of acute coronary artery disease 2 months after surgery.

## Background

Hepatoid carcinoma is an extremely rare form of neoplasm. Its cellular structure resembles that of a hepatocellular carcinoma (HCC). Hepatoid carcinoma (HC) has been described in various organs and systems such as the gastrointestinal tract, testes, ovaries, and lungs [1]. We present a case of a HC of the pancreatic head in a 78-year-old male patient.

## Case presentation

A 78-year-old male patient with diabetes underwent an annual abdominal ultrasound scan. He had no complaints, and his diabetes was controlled well. The patient denied experiencing weight loss, nausea, jaundice, fever, and other diseases. He was a non-smoker without increased alcohol consumption.

**Fig. 1:**
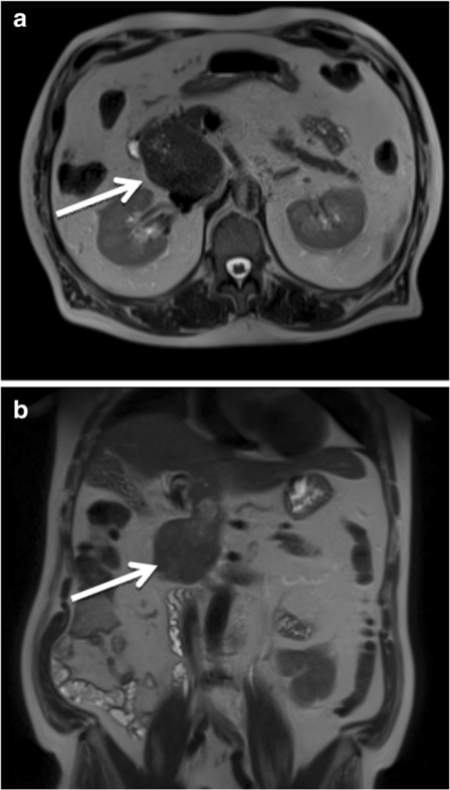
MRI scan of the discovered mass which compresses the large intraabdominal vessels (arrow)1**a** transverse section of the tumor 1**b** frontal section

**Fig. 2:**
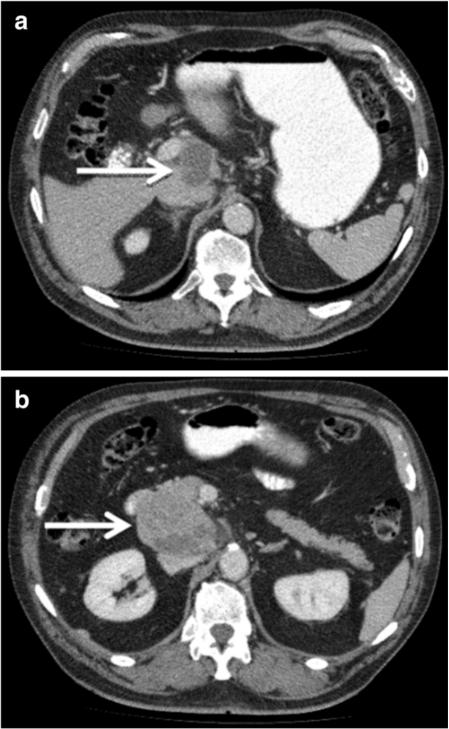
CT scan showing a large hypodense mass between the liver and the pancreas head (2**a** and 2**b**)

Physical examination showed a soft, non-tender, and non-distended abdomen with no masses and normal bowel sounds. Contrast-enhanced ultrasound revealed an 8 × 6 cm large, hypervasculated tumor in segment I of the liver with infiltration in the pancreas. An MRI showed an 8-cm large, hypointense mass with a probable hepatic origin and abnormal growth retroperitoneal to the pancreatic head (Fig. 1a, b). The large vessels seemed to be compressed but not infiltrated. Laboratory studies revealed a monoclonal gammopathy of undetermined significance (MGUS) with urine protein excretion at 69 mg/g creatinine. The tumor markers AFP, CA 19-9, and CEA were not elevated. Abdominal and thoracic CT-scans with contrast enhancement confirmed the 8-cm large mass between the pancreatic head and an additional hemangioma (Fig. 2a, b) in segment 8. There was no evidence of metastases.

**Fig. 3:**
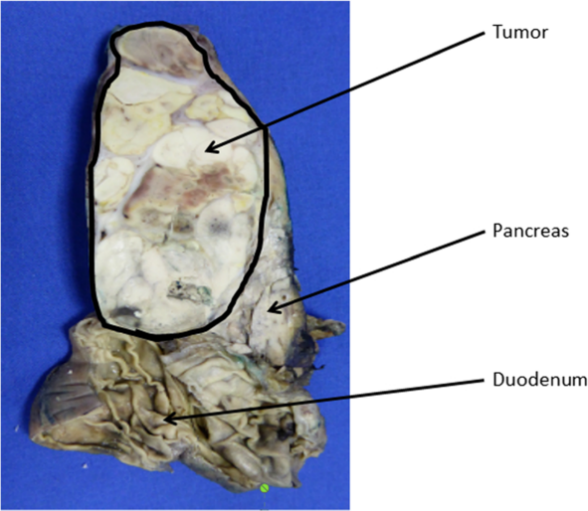
Macroscopy: a well-encapsulated tumor in the head of the pancreas. There is no infiltration into the duodenum

**Fig. 4:**
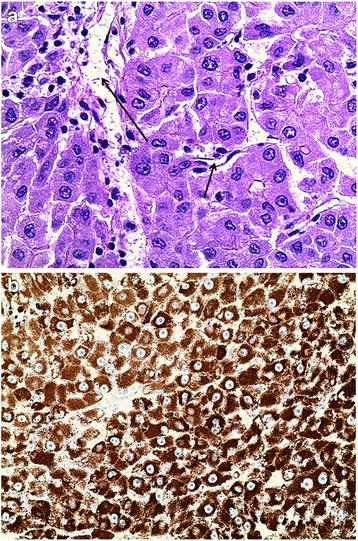
**a** The tumor is composed of polygonal cells with granular eosinophilic cytoplasm, centrally located nuclei, and prominent nucleoli. Capillary (*arrow*) (H&E, ×400). **b** Immunohistochemical staining of the tumor for hepatocyte specific antigen (Hep Par 1)—cytoplasm of neoplastic cells is strongly positive whereas the centrally located nuclei with prominent nucleoli are negative (H&E, ×200)

It was assumed that the monoclonal IgA gammopathy was not associated with the current disease. To determine the nature of the retroperitoneal mass, a CT-guided biopsy was performed forwarding only fibrous cells. An exploratory laparoscopy was done next, and a laparoscopic ultrasound showed a homogenous mass behind the pancreatic head separated from the liver. The mesentery root was not infiltrated. Core needle biopsies showed “epithelial tumor of an unknown dignity”; further classification was not possible. As a malignant tumor was still strongly suspected, the patient underwent surgical resection (Whipple procedure) and the tumor was removed completely. The further clinical course was uneventful. The final pathology report described hepatoid carcinoma of the pancreatic head with a size of 12 cm without infiltration of the duodenal wall (pathological T3, N0 (0/10), L0, V0, R0, stage IIB) (Fig. 3). The excised specimen displayed an encapsulated, lobulated, well-differentiated epithelial tumor with tumor cells growing in a solid pattern, characterized by a large amount of cytoplasm, enlarged round nuclei and nucleoli. Imunnohistochemical staining of the tumor for hepatocyte specific antigen (Hep Par 1) showed a strongly positive reaction in the cytoplasm of the neoplastic cells while the centrally located nuclei remained negative. Tumor blood was supplied by a well-developed intercellular capillary network, identified by a positive CD34 reaction (Fig. 4a, b).

The postoperative recovery was slow due to gastric atony, which required parenteral nutrition, and a tendency to hypoglycemia.

Considering the age and clinical condition of the patient together with the stage of the disease, no further therapy was recommended by the tumor board and he was discharged home. Regular follow-up was to take place. Two months after surgery, the patient suddenly suffered an acute heart attack and was pronounced dead upon arrival at the emergency room.

### Discussion

Hepatoid carcinoma is an extremely rare form of neoplasm. The tumor has been found in various organs, including the gastrointestinal tract, testes, and ovaries [2–5]. Recently, Motooka and colleagues reported a new case of a hepatoid carcinoma of the lung [6]. The diagnosis is complex, since the tumor can clinically mimic other neoplasms and the laboratory results are often unspecific as in this reported case. Discriminating from a HCC and neuroendocrine tumors using medical imaging is challenging. A CT- or an endoscopy-guided fine needle biopsy should be performed when possible.

The cellular structure of the tumor resembles that of a HCC. Typical histological features are islands of large tumor cells growing in a trabecular or perisinusoidal pattern, displaying an eosinophilic cytoplasm and round nuclei as well as prominent nucleoli [1, 7, 8]. It is assumed that HC derives from ectopic liver tissue because both the pancreas and liver have a common embryologic origin—the foregut endoderm [7]. Some tumor cells express AFP, hepatocyte antigen, and cytokeratin. AFP [9] and PIVKA-II [10] have been suggested as a tumor marker for the disease. Staining for hepatocellular differentiation is typically positive, including hepatocyte paraffin 1 (HepPar1), polyclonal CEA (canalicular), and CD10 (canalicular). The differential diagnosis of hepatoid carcinoma includes acinar cell carcinoma and pancreatoblastoma, both can express AFP, as well asintraductaloncocytic papillary neoplasm, which consistently stains for HepPar1, but it does not possess true hepatocellular differentiation [11]. In order to exclude a metastatic HCC, some authors suggest the examination of two specific hepatocyte-transporters (bile salt export pump (BSEP) and multidrug-resistance protein 3 (MDR3)) which are not usually expressed by HC tumor cells [12].

To date, only 26 cases of pancreatic HC have been reported in the literature. The patients’ age ranged from 21 to 80 years. Most of them were male (16M/10F). Thirteen patients were either asymptomatic or suffered from unspecific abdominal pain. Four patients however showed jaundice. All patients underwent surgery and/or chemotherapy. Nine of them died despite therapy within 2 to 101 months after diagnosis. In 7 cases, extensive hepatic metastases were noticed. There are reports which describe the tumor as highly aggressive with a tendency for early hepatic metastases [13]. Most authors report pure HC [14–16]. These tumors have the highest incidence of metastasis and the worst prognosis. In some cases, the tumor has been found associated with a neuroendocrine carcinoma [9, 17, 18]. The latter showed a longer survival and sensitivity to radiochemotherapy [9]. Three of the cases describe tumors with primarily hepatocellular differentiation [19–21], and the authors suspect the origin of the tumors to be ectopic liver tissue. In those cases, the tumors were not metastatic and the patients had improved survival. Association of HC [7], with a serous microcystic cystadenoma, has been reported in a single patient who remained free from metastases and showed no recurrence during the 8 months of follow-up. One case of Papilla vateri HC was also reported [22].

No standard therapy has been established due to the tumor rarity and the lack of evidence-based data. Tumor resection is, if possible, always recommended. Neoadjuvant and adjuvant chemotherapy can be considered; however, standard therapy usually used for pancreas neoplasm with gemcitabine is inefficient. The XELOX protocol with oxaliplatin and capecitabine led to remission in some cases [7]. A short-term regression can be achieved with sorafenib [13, 15]. Unfortunately, the long-term outcome of the current therapy is poor. Further studies and new treatment approaches are needed to determine the biological behavior of the tumor and its sensitivity to chemotherapeutical agents.

## Conclusions

We have presented a case of a non-metastatic HC of the pancreatic head. HC is an extremely rare form of neoplasm, which has morphological and immunohistochemical features similar to those of HCC. The patient was only treated operatively, owing to his clinical condition and considering the stage of the tumor. He died of a sudden coronary event 2 months after the operation.

## Abbreviations

MRI, magnetic resonance imaging; MGUS, monoclonal gammopathy of undetermined significance; AFP, alpha fetoprotein; CA 19-9, carbohydrate antigen/cancer antigen 19-9; CEA, carcinoembryonic antigen; CT, computed tomography; IgA, immunoglobuline A; HCC, hepatocellular carcinoma; H&E, hematoxylline/eosine stain; PIVKA-II, prothrombin induced by vitamin K absence-II
